# Relationships between cardiorespiratory fitness/muscular strength and ^18^F-fluorodeoxyglucose uptake in brown adipose tissue after exposure to cold in young, sedentary adults

**DOI:** 10.1038/s41598-019-47918-7

**Published:** 2019-08-05

**Authors:** Borja Martinez-Tellez, Guillermo Sanchez-Delgado, Francisco J. Amaro-Gahete, Francisco M. Acosta, Jonatan R. Ruiz

**Affiliations:** 1 PROFITH (PROmoting FITness and Health through Physical Activity) Research Group, Sport and Health University Research Institute (iMUDS), Department of Physical Education and Sport, Faculty of Sport Sciences, University of Granada, Granada, Spain; 2 Department of Medicine, division of Endocrinology, and Einthoven Laboratory for Experimental Vascular Medicine, Leiden University Medical Center, Leiden, The Netherlands; 3 Department of Medical Physiology, School of Medicine, University of Granada, Granada, Spain

**Keywords:** Endocrine system and metabolic diseases, Biomarkers

## Abstract

Humans have metabolically active brown adipose tissue (BAT). However, what is the relation between exercise or physical activity with this tissue remains controversial. Therefore, the main aim of the present study is to examine whether cardiorespiratory fitness and muscular strength are associated with brown adipose tissue (BAT) volume and activity after exposure to cold in young, sedentary adults. Cardiorespiratory fitness was determined in 119 young, healthy, sedentary adults (68% women, age 21.9 ± 2.1 years, body mass index 25 ± 4.8 kg/m^2^) via the maximum treadmill exercise test, and their muscular strength assessed by the handgrip strength test and the 1-repetition maximum bench and leg press tests. Some days later, all subjects were exposed to 2 h of personalized exposure to cold and their cold-induced BAT volume and activity determined by a combination of ^18^F-fluorodeoxyglucose (^18^F-FDG) positron emission tomography and computed tomography scan. Cardiorespiratory fitness was associated with neither the BAT volume nor BAT activity (P ≥ 0.05). However, handgrip strength with respect to lean body mass was positively (though weakly) associated with BAT activity as represented by the ^18^F-FDG mean standardised uptake value (SUV) (β = 3.595, R^2^ = 0.039, P = 0.031) and SUVpeak value (β = 15.314, R^2^ = 0.037, P = 0.035). The above relationships remained after adjusting for several confounders. No other associations were found. Handgrip strength with respect to lean body mass is positively associated with BAT activity (SUVmean and SUVpeak) in young adults after exposure to cold - but only weakly. Further studies are needed to reveal the relationship between muscular fitness and human BAT characteristics.

## Introduction

Humans have metabolically active brown adipose tissue (BAT)^1–3^. Based on the ability of BAT to increase energy expenditure in mice, the activation of human BAT has been proposed as a means of treating adiposity^4^. BAT may also act as an endocrine organ, helping to control metabolic homeostasis. However, humans have relatively less BAT than mice^5^; its involvement in human metabolism may therefore be different.

Feasible strategies for activating and recruiting BAT are needed. Cold has been shown the main BAT-activating stimulus in humans^2,6,7^, but some studies suggest that certain drugs and dietary components^8,9^ may also provide such a stimulus. Whether exercise activates BAT or induces its recruitment is uncertain^9^. Exercise is an effective aid in preventing many physical^10^ and mental^11^ diseases^12^, including adiposity, but its effect on BAT remains controversial^13–15^. Case-control studies have shown that the BAT ^18^F-fluorodeoxyglucose (^18^F-FDG) uptake of endurance-trained men^16^ and women^17^ [high levels of maximum oxygen uptake (VO2max)] is lower than that of sedentary and non-athlete controls [low VO2max]. Exercise interventions have recorded contradictory results regarding BAT metabolism, although some authors report they may increase the expression of ‘fat browning’ genes in the abdominal subcutaneous adipose tissue^18,19^. Dinas *et al*.^20^ showed BAT ^18^F-FDG uptake (i.e., BAT activity) to be positively related to physical activity levels as measured by questionnaire, although this study suffered from several limitations^21^. In contrast, our group reported physical activity levels (measured via accelerometry) not to be associated with BAT volume or activity in a sedentary cohort of young, healthy adults^22^.

Exercise is a structured, planned and repetitive subtype of physical activity designed to improve or maintain physical fitness^23^. Physical fitness is a powerful marker of health and a better predictor of morbidity and mortality^24,25^ than physical activity, and cardiorespiratory fitness and muscular strength are two of its main components^25^. Cardiorespiratory fitness reflects the overall capacity of the cardiovascular and respiratory systems, and therefore the ability to carry out prolonged exercise. Cardiorespiratory fitness is measured by a VO2max test. It also provides reliable, prognostic information about the overall risk of illness and death in both men and women across a wide age range^26^. In addition, several prospective studies have shown that muscular strength, i.e., the ability of a muscle to develop a maximum contractile force against a resistance, to be inversely associated with all-cause mortality^24^. Henriksson *et al*.^27^ recently showed that high levels of muscular strength were associated with a lower risk of eventually needing a disability pension. Whether cardiorespiratory fitness and muscle strength are related to BAT volume and activity remains to be investigated, but based on available evidence, it can be hypothesized that cardiorespiratory fitness is negatively associated with BAT volume and activity.

The aim of the present work was to study the association of cardiorespiratory fitness and muscular strength with BAT volume and activity (determined via ^18^F-FDG uptake) after exposure to cold in young, sedentary adults.

## Materials and Methods

### Research design and participants

This cross-sectional study was performed within the framework of the ACTIBATE project^28^. All assessments were made in Granada (Spain) during the months of October, November and December 2015 and 2016. The study subjects were 119 young adults, 38 of whom were men. All underwent a comprehensive medical examination and reported themselves to be sedentary (<20 min moderate-vigorous physical activity on <3 days/week), reported a stable body weight over the last 3 months (<3 kg change), were not exposed to cold regularly, did not smoke, and took no medication. None suffered from cardiometabolic disease. The study was performed in accordance with current ethical guidelines (Declaration of Helsinki, as revised in 2013) and were approved by the Human Research Ethics Committee of the University of Granada (n°924) and that of the *Servicio Andaluz de Salud*. All participants gave their written, informed consent to be included.

### Procedures

#### Body composition and anthropometric variables

Lean body mass (LBM) and fat body mass were determined using a Hologic Discovery Wi dual energy x-ray absorptiometer (DXA) (Hologic, Massachusetts, USA). Body weight and height were measured using a SECA model 799 electronic column scale and stadiometer (SECA, Hamburg, Germany). Body mass index (BMI) and lean mass index (LMI) were calculated as body weight/LBM divided by height squared.

#### Cardiorespiratory fitness

Subjects arrived at our test centre after fasting for 3–5 h. They had performed no vigorous exercise in the previous 48 h, nor moderate exercise in the previous 24 h, and had consumed neither coffee nor tea in the latter period.

Cardiorespiratory fitness was determined using a treadmill maximum exercise test employing an H/P/Cosmos Pulsar treadmill (H/P/Cosmos Sports & Medical GmbH, Nussdorf-Traunstein, Germany), following the modified Balke protocol^28^. This involved a warm-up of 1 min at 3 km/h, followed by 2 min at 4 km/h. In the fourth minute, the speed of the treadmill was increased to 5.3 km/h with the slope at 0%. Every minute thereafter the treadmill slope was increased by 1% until the subjects became exhausted. Respiratory gas exchange was monitored during the test by indirect calorimetry using a CPX Ultima CardioO2 gas exchange analysis system (Medical Graphics Corp, St Paul, MN, USA) equipped with a model 7400 plastic facemask (Hans Rudolph Inc., Kansas City, MO, USA) and a preVent™ metabolic flow sensor (Medical graphics Corp, St Paul, MN, USA)^29^. VCO_2_ was measured using a non-dispersive infra-red sensor, and VO_2_ using a galvanic fuel cell^29^. Maximum oxygen volume (VO_2_max) was defined as a respiratory exchange ratio of ≥1.1, having reached a VO_2_ plateau (change of <100 ml/min over three consecutive 10 s intervals), and a heart rate within 10 beats/min of the age-predicted maximum (209–0.73 × age^30^. Time to exhaustion was measured in seconds). Since aerobic performance depends on body mass and composition^27^, VO_2_max was represented in absolute terms, relative to body mass, and relative to LBM.

#### Muscular strength

Muscular strength was measured by three tests: the handgrip strength test, and the 1 repetition maximum (1-RM) bench and leg press tests.

Handgrip strength test. This was assessed using a Takei 5401 digital Grip-D hand dynamometer (Takei, Tokyo, Japan)^31^. Subjects stood with the shoulder of the tested side slightly abducted and the corresponding arm hanging straight down (not touching the rest of the body [~10° separation]) with no inflexion of the elbow. They were then asked to squeeze the grip gradually and continuously, and encouraged to do their best while performing the test. Their maximum strength was recorded automatically by the dynamometer. Each participant performed the test twice, alternating between hands with 1 min rest between attempts. The same grip span was used by the male subjects, but was adjusted to suit each female subject^31^. The highest values (in kg) were recorded for analysis. Results were represented in absolute terms, relative to body mass, and relative to LBM.

1-RM bench and leg press tests. Upper and lower body strength were assessed via a supine bench press test and a leg press test using a KEISER® Power rack a KEISER® Air 300 pneumatic resistance machine respectively (Keiser, Fresno, CA, USA). 1-RM measurements were not made directly since the subjects were sedentary, but by making use of the Wathen equation - a valid means of estimating 1-RM values for the upper and lower body^32^ in untrained individuals. In order to use this equation, subjects lifted the maximum weight they could lift a maximum number of 1–10 times in both tests:

$$1 \mbox{-} {\rm{RM}}=\frac{Weight\,lifted\,per\,repetition\,(kg)}{(48.8+53.8{e}^{-0.075\times numberofrepetitions})/100}$$

All subjects were allowed three attempts to provide the required lift data, returning on a different day to try again if they failed to do so. All subjects performed several lifts with no weight to familiarise themselves with the exercise. If they realised they would be able to perform more than 10 lifts with a particular weight, they stopped and rested for at least 5 min before making another attempt with a heavier weight. When subjects performed <10 repetitions at their maximum strength capacity, the exercise was deemed concluded. 1-RM values for both the bench and leg press tests were recorded in absolute terms, relative to body mass, and relative to LBM.

#### Positron emission tomography/computed tomography (PET/CT)

The cooling protocol used and the quantification of the BAT volume and activity were as previously reported^22,33,34^. Briefly, subjects sat in a cool room (19.5–20 °C) wearing a water-perfused cooling vest (Polar Products Inc., Stow, OH, USA). The water temperature was reduced from 16.6 °C at ~2.2 °C per 10 min until they began shivering. After 48–72 h had elapsed they went to the *Hospital Virgen de las Nieves*, where they were again placed in a cool room (19.5–20 °C) and wore the same cooling vest but with the water temperature set ~4 °C above their earlier shivering threshold test result for 2 h. After the first hour the subjects received an injection of ^18^F-FDG (~185 MBq) and the water temperature was increased by 1 °C to avoid visually detectable shivering. One hour later they were subjected to PET/CT using a Siemens Biograph 16 PET/CT scanner (Siemens, Erlangen, Germany), scanning two BEDs from the atlas vertebra to thoracic vertebra 6 (approximately).

The BAT volume and BAT ^18^F-FDG activity were then determined following recent recommendations^35^ using the Beth Israel plugin for the FIJI program^34^. This required the determination of: 1) the number of pixels in the region of interest (ROI) with a radiodensity range of −190 to −10 Hounsfield Units; and 2) individualized, standardized threshold ^18^F-FDG uptake values (SUV) [1.2/(lean body mass/body mass)]^35^. BAT volume was determined as the number of pixels in the above range with an SUV value above the SUV threshold. BAT activity was determined with respect to the mean SUV (SUVmean: the mean quantity of ^18^FDG in the above same pixels) and peak SUV (SUVpeak; the mean of the three highest ^18^F-FDG contents in three pixels within a volume of <1 cm^3^). The SUVpeak for the descending aorta (reference tissue) was also determined, and for several skeletal muscles between the atlas vertebra and thoracic vertebra 4 using a single ROI from one slice (image) of the paracervical, sternocleidomastoid, scalene, longus colli, trapezius, parathoracic, supraspinatus, subscapular, deltoid, pectoralis major, and triceps brachii muscles from both the left and right side of the body^6,36^. An ROI of white adipose tissue (WAT) was also chosen in the dorsocervical area since ^18^F-FDG uptake can be greater here than elsewhere^37^. A mean for the SUVpeak values recorded for all the examined muscles, on both sides, was calculated to provide a representative value for all skeletal muscle ^18^F-FDG uptake. Mean SUVpeak values for the different skeletal muscle groupings were also calculated^36^.

#### Statistical analysis

Data are presented as means ± standard deviations unless otherwise stated. Univariate linear regression (Model 1) was used to examine the associations of cardiorespiratory fitness and muscular strength variables with BAT volume and activity, as well as with ^18^F-FDG uptake by the mentioned skeletal muscles (dependent variables). Multiple linear regression was used to test these associations after adjusting for the date when PET/CT was performed (Model 2), and for the date when PET/CT was performed plus sex (Model 3). Since sex had no effect on any of the associations (all P ≥ 0.05), the results for all subjects were analysed together. All calculations were performed using the Statistical Package for the Social Sciences v.22.0 (IBM SPSS Statistics, IBM Corporation). Significance was set at P < 0.05.

## Results

**Figure 1:**
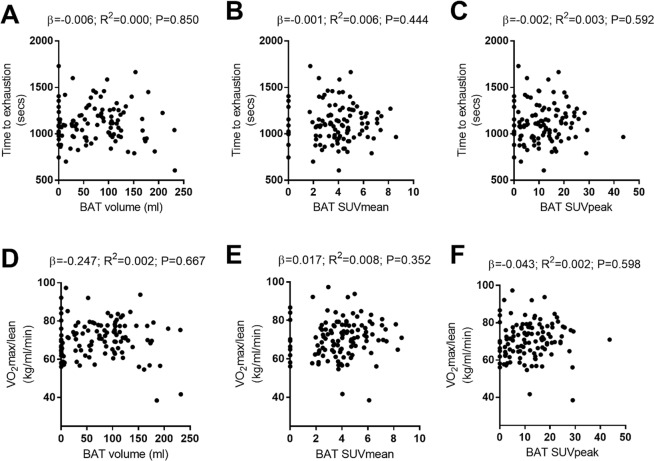
Associations between cardiorespiratory fitness (VO_2_max, ml/kg lean body mass (LBM)/min, and time to exhaustion) and brown adipose tissue (BAT) volume and activity after personalised cold exposure. N = 98 participants. β and P from univariate linear regression analysis. β = non-standardised coefficients; R^2^ = explained variance; SUV = standardized uptake value.

Table 1 summarises the subjects’ personal characteristics and test results. Figure 1 shows the association between the cardiorespiratory fitness (treadmill test) values (i.e., the time to exhaustion and VO_2_max relative to LBM) and BAT volume/activity. No association was seen between time to exhaustion and BAT volume, BAT SUVmean or BAT SUVpeak (Fig. 1A: β = −0.006, R^2^ = 0.000, P = 0.850; β = −0.001, R^2^ = 0.006, P = 0.444; and β = −0.002, R^2^ = 0.003, P = 0.592, respectively). Neither was any association seen between VO_2_max and BAT volume, BAT SUVmean or BAT SUVpeak (Fig. 1B: β = −0.247, R^2^ = 0.002, P = 0.667; β = −0.017, R^2^ = 0.008, P = 0.352; and β = −0.04, R^2^ = 0.002, P = 0.598, respectively). The lack of association persisted after adjusting for the date when PET/CT was performed (Model 2), and the date when the PET/CT was performed plus sex (Model 3) (Table 2). Table 2 also shows the lack of association between cardiorespiratory fitness and BAT variables when the results were analysed in absolute terms and relative to body mass. However, multiple regression with Models 2 and 3 showed VO_2_max relative to body weight to be negatively associated with the BAT volume (β = −1.363, R^2^ = 0.215, P = 0.025 and β = −1.731, R^2^ = 0.244, P = 0.006, respectively).

**Table 1:** Characteristics of the study subjects.

	All sample	Men	Women
	Mean SD	Mean SD	Mean SD
N (% men)	119 (31.9%)	38	81
Age (years old)	21.9 ± 2.1	22.1 ± 2.2	21.8 ± 2.1
Body mass index (kg/m^2^)	25.0 ± 4.8	27.6 ± 5.7	23.7 ± 3.9
Lean mass index (kg/m^2^)	14.6 ± 2.4	17.2 ± 2.1	13.3 ± 1.4
Fat mass percentage (%)	36.3 ± 7.2	31.7 ± 7.8	38.4 ± 5.9
Handgrip strength (kg)	31.2 ± 7.8	40.0 ± 6.7	27.0 ± 3.8
1-RM leg press (kg)	200.6 ± 69.4	281.3 ± 50.9	162.7 ± 36.9
1-RM bench press (kg)	31.5 ± 14.9	49.7 ± 12.0	23.0 ± 5.5
VO_2_max (ml/kg/min) body mass	41.4 ± 7.9	44.2 ± 9.6	40.1 ± 6.6
BAT volume (ml)	73.8 ± 58.7	92.2 ± 68.1	65.2 ± 51.9
BAT SUVmean	3.91 ± 1.91	3.64 ± 1.29	4.03 ± 2.13
BAT SUVpeak	11.79 ± 8.31	11.45 ± 7.50	11.95 ± 8.71
All skeletal muscle SUVpeak	0.81 ± 0.20	0.81 ± 0.18	0.81 ± 0.21
Descending aorta SUVpeak	1.57 ± 0.33	1.66 ± 0.35	1.52 ± 0.32

Data are presented as mean ± SD. BAT brown adipose tissue; 1-RM = 1 maximum repetition test; SUV = standardized uptake value; VO_2_max = maximum volume of oxygen consumed.

**Table 2:** Associations between cardiorespiratory fitness variables and brown adipose tissue (BAT) variables.

	BAT volume (ml)			BAT SUVmean			BAT SUVpeak		
	β	R^2^	P	β	R^2^	P	β	R^2^	P
**MODEL 1**									
Time to exhaustion (s)	−0.006	0.000	0.850	−0.001	0.006	0.444	−0.002	0.003	0.592
VO_2_max (ml/min)	0.013	0.024	0.095	0.000	0.013	0.217	−0.001	0.004	0.484
VO_2_max relative to LBM (ml/kg/min)	−0.247	0.002	0.667	0.017	0.008	0.352	0.043	0.002	0.598
VO_2_max relative to body weight (ml/kg/min)	−1.274	0.028	0.057	−0.026	0.011	0.235	−0.100	0.009	0.289
**MODEL 2**									
Time to exhaustion (s)	0.005	0.135	0.846	−0.001	0.144	0.101	−0.005	0.151	0.147
VO_2_max (ml/min)	0.009	0.191	0.182	0.000	0.170	0.073	−0.001	0.172	0.215
VO_2_max relative to LBM (ml/kg/min)	−0.641	0.189	0.224	0.006	0.147	0.719	−0.008	0.160	0.912
VO_2_max relative to body weight (ml/kg/min)	−1.363	0.215	**0**.**025**	−0.029	0.157	0.160	−0.111	0.168	0.199
**MODEL 3**									
Time to exhaustion (s)	−0.004	0.145	0.884	−0.001	0.168	0.285	−0.004	0.159	0.273
VO_2_max (ml/min)	−0.003	0.212	0.753	0.000	0.171	0.343	−0.001	0.172	0.359
VO_2_max relative to LBM (ml/kg/min)	−0.541	0.219	0.299	0.004	0.165	0.828	−0.014	0.166	0.851
VO_2_max relative to body weight (ml/kg/min)	−1.731	0.244	**0**.**006**	−0.016	0.187	0.432	−0.079	0.180	0.379

Model 1: Unadjusted. Model 2: adjusted by date when positron emission tomography/computed tomography (PET/CT) was performed. Model 3: adjusted by date of PET/CT and sex. β = non-standardised coefficients; BM = body mass; R^2^ = explained variance; SUV = standardised uptake value; LBM: lean body mass; VO_2_max = maximum volume of oxygen consumed. n = 98 subjects.

**Figure 2:**
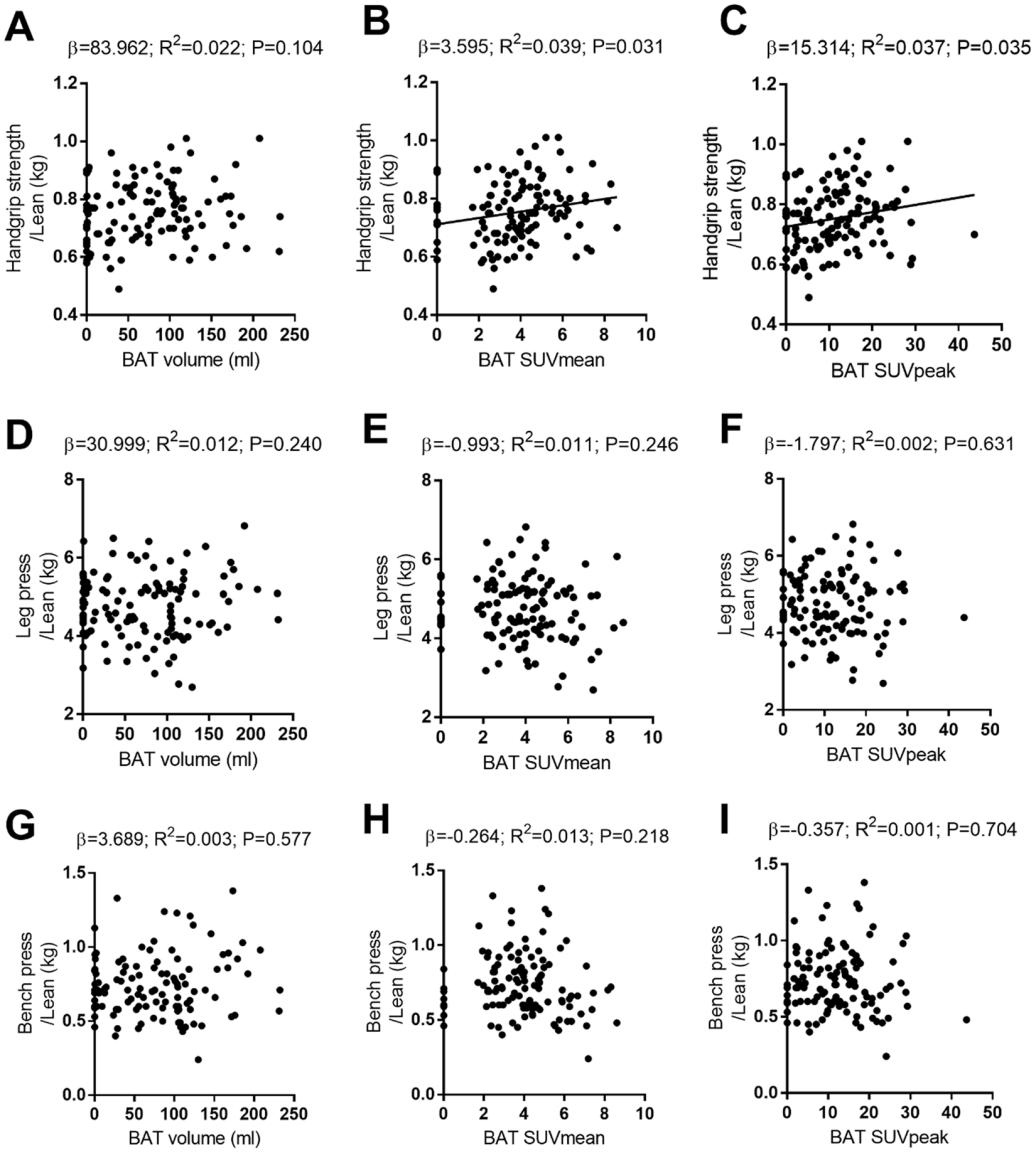
Associations of handgrip strength and leg and bench press results, both relative to lean body mass (LBM), with brown adipose tissue (BAT) volume and activity. N = 119 participants. β and P from univariate linear regression analysis. β = non-standardised coefficients; R^2^ = explained variance; SUV = standardized uptake value.

A panel of experts recently reported that BAT ^18^F-FDG uptake depends strongly on LBM^35^. The results for muscular strength relative to LBM were therefore paid special attention in analyses. Figure 2 shows the association of muscular strength (i.e., as determined by the handgrip strength/LBM, leg press/LBM and bench press/LBM results) with BAT volume and activity, as determined by univariate linear regression. No association was seen between handgrip strength and BAT volume (β = 83.962, R^2^ = 0.022, P = 0.104; Fig. 2A), whereas it was positively associated with BAT SUVmean and BAT SUVpeak (β = 3.595, R^2^ = 0.039, P = 0.031; and β = 15.314, R^2^ = 0.037, P = 0.035; Fig. 2B,C). No association was seen between the leg press test results with respect to LBM and BAT volume or activity (all P ≥ 0.218, Fig. 2D–F), or between the bench press test results relative to LBM and BAT volume or activity (all P ≥ 0.240; Fig. 2G–I). The results persisted after controlling for the date when the PET/CT was performed using Model 2, and for the date when the PET/CT was performed plus sex using Model 3 (Table 3). Table 3 also shows that all muscular strength values in absolute terms were positively associated with the BAT volume in the Model 1 and 2 regressions, while in Model 3 only handgrip strength remained positively and significantly associated with BAT volume. No associations were found between muscular strength relative to body weight and BAT volume or activity (Table S1).

**Table 3:** Associations between muscular fitness variables and brown adipose tissue variables.

	BAT volume (ml)			BAT SUVmean			BAT SUVpeak		
	β	R^2^	P	β	R^2^	P	β	R^2^	P
**MODEL 1**									
Handgrip strength (kg)	2.344	0.096	**0**.**001**	−0.009	0.001	0.708	0.046	0.002	0.642
Leg press (kg)	0.156	0.034	**0**.**044**	−0.004	0.026	0.078	−0.009	0.005	0.440
Bench press (kg)	0.781	0.039	**0**.**031**	−0.016	0.016	0.172	−0.027	0.002	0.595
Handgrip strength/LBM (kg)	83.962	0.022	0.104	3.595	0.039	**0**.**031**	15.314	0.037	**0**.**035**
Leg press/LBM (kg)	30.999	0.012	0.240	−0.993	0.011	0.246	−1.797	0.002	0.631
Bench press/LBM (kg)	3.689	0.003	0.577	−0.264	0.013	0.218	−0.357	0.001	0.704
**MODEL 2**									
Handgrip strength (kg)	2.107	0.247	**0**.**001**	−0.016	0.142	0.452	0.013	0.149	0.889
Leg press (kg)	0.147	0.200	**0**.**038**	−0.005	0.168	**0**.**044**	−0.010	0.156	0.340
Bench press (kg)	0.653	0.197	**0**.**049**	−0.020	0.163	0.067	−0.045	0.156	0.344
Handgrip strength/LBM (kg)	70.724	0.185	0.135	3.211	0.169	**0**.**040**	13.568	0.178	**0**.**045**
Leg press/LBM (kg)	3.257	0.172	0.591	−0.277	0.153	0.166	−0.414	0.151	0.633
Bench press/LBM (kg)	21.807	0.175	0.368	−1.275	0.157	0.110	−3.057	0.155	0.379
**MODEL 3**									
Handgrip strength (kg)	2.532	0.249	**0**.**011**	0.019	0.156	0.567	0.147	0.159	0.317
Leg press (kg)	0.055	0.206	0.643	−0.006	0.169	0.149	−0.013	0.156	0.453
Bench press (kg)	0.099	0.205	0.870	−0.023	0.163	0.248	−0.068	0.156	0.443
Handgrip strength/LBM (kg)	67.768	0.220	0.145	3.277	0.186	**0**.**035**	13.701	0.182	**0**.**043**
Leg press/LBM (kg)	−3.875	0.207	0.565	−0.184	0.158	0.415	−0.205	0.152	0.836
Bench press/LBM (kg)	−28.079	0.210	0.389	−0.928	0.159	0.396	−2.936	0.155	0.538

Model 1: Unadjusted. Model 2: adjusted by date when positron emission tomography/computed tomography (PET/CT) was performed. Model 3: adjusted by date of PET/CT and sex. β = non-standardised coefficients; BM = body mass; R^2^ = explained variance; SUV = Standardised uptake value; LBM: lean body mass; VO_2_max = maximum volume of oxygen consumed. n = 119 subjects.

No association was seen between cardiorespiratory fitness with respect to LBM and ^18^F-FDG uptake by the skeletal muscles, dorsocervical adipose tissue, or the reference tissue (Table S2). Nor was any seen between muscular fitness outcomes with respect to LBM and ^18^F-FDG uptake by the same tissues (Table S3).

## Discussion

The present results show that cardiorespiratory fitness is not associated with BAT volume or activity after cold exposure in young, sedentary adults. However, handgrip strength with respect to LBM was positively and significantly associated with BAT activity (SUVmean and SUVpeak). Muscular strength variables in absolute terms were also positively and significantly associated with BAT volume, yet the associations disappeared once muscular strength was relative to body weight.

To date, two case-control studies involving a group of sedentary persons/non-athletes and endurance-trained men^16^ and women^17^ have examined the differences between cardiorespiratory fitness and human BAT characteristics. Both revealed the trained persons, who had higher levels of cardiorespiratory fitness, to have a smaller BAT volume and to show a lower BAT ^18^F-FDG uptake. In the present study, however, no association was found between cardiorespiratory fitness variables and BAT volume or activity.

Human studies on BAT volume and activity have largely focused on how the latter are affected by endurance training^9,38^. Less attention has been paid to other types of exercise, such as resistance training, which induces different physiological adaptations^39^. For instance, this latter type of exercise is an anabolic stimulus for skeletal muscle, and has been shown to increase muscle mass^40^ and energy expenditure^41^. The presently recorded positive (though weak) association between handgrip strength and BAT activity suggests a link between muscular strength and BAT metabolism. Resistance training can improve handgrip strength^42^, but whether resistance training can also modify BAT ^18^F-FDG uptake remains to be seen. Future studies should focus on the type of exercise (endurance vs. resistance), intensity (moderate vs. vigorous), and subject training status (untrained vs. trained individuals)^9^. Interestingly, in the present work no such positive association between the other muscular fitness variables measured (1-RM for bench and leg press tests) and BAT ^18^F-FDG uptake was detected, despite the fact that the Pearson correlation coefficients between handgrip strength and 1-RM estimates for both the bench and leg press were above r = 0.4 (data not shown). However, this type of finding is not entirely novel: several studies have shown handgrip strength to be the muscular strength variable that best predicts mortality^24,43,44^. The differences observed in the association between with BAT ^18^F-FDG uptake and the handgrip strength and 1-RM bench and leg press results might be explained in that, while the technique involved in the handgrip strength test is used by all people in their normal life (i.e., for shaking hands, carrying bags, gripping things, etc.), the bench and leg press tests can only be performed once the techniques required have been learned. All the present subjects were sedentary and for the vast majority this was their first experience with these exercises. The ability to perform them is thus influenced by a learning process.

WAT can transdifferentiate into brown-like cells via a process commonly named as browning^45^; the cells produced are known as known as BRITE (brown-in-white) cells. Animals studies have shown that endurance exercise may induce browning of the WAT more than activation of the regular BAT^38^. Whether exercise is able to induce browning in humans is unclear^9^. Vosselman *et al*.^16^ reported finding no differences in abdominal subcutaneous WAT browning markers between trained people and their sedentary counterparts. Under different circumstances, however, Dinas *et al*.^46^ observed that people reporting higher levels of physical activity had higher browning marker levels in this same WAT. However, no studies investigating how physical fitness is related to browning in humans have been published, so little is known about this particular relationship.

The present study suffers the limitation of its cross-sectional design, which precludes the establishment of cause-effect relationships. The positive correlation between handgrip strength and BAT activity is weak, although it seems to persist in different regression analysis. Further, ^18^F-FDG uptake (as a proxy for glucose uptake) may not represent the whole story; BAT consumes more fatty acids than it does glucose^47^. Repeating these tests using others tracers of BAT activity^48^, and in other populations, would be of interest. Moreover, we analysed the correlation between physical fitness with BAT ^18^F-FDG uptake measured after cold exposure, it would be of interest to test this association when BAT is measured at thermoneutral conditions or with other nuclear medicine techniques.

## Conclusions

Cardiorespiratory fitness is not associated with human BAT activity (measured as ^18^F-FDG uptake), and handgrip strength relative to LBM was the only variable positively associated with BAT activity (SUVmean and SUVpeak) - but weakly. Further studies are needed to examine the relationship between muscular fitness and human BAT activity and WAT browning.

## Supplementary information

Extra analyses

## References

[1] Virtanen KA (2009). Functional brown adipose tissue in healthy adults. N. Engl. J. Med..

[2] van Marken Lichtenbelt WD (2009). Cold-activated brown adipose tissue in healthy men. N. Engl. J. Med..

[3] Cypess AM (2009). Identification and importance of brown adipose tissue in adult humans. N. Engl. J. Med..

[4] Whittle AJ, López M, Vidal-Puig A (2011). Using brown adipose tissue to treat obesity - the central issue. Trends Mol. Med..

[5] Carpentier AC (2018). Brown Adipose Tissue Energy Metabolism in Humans. Front. Endocrinol. (Lausanne)..

[6] Hanssen MJW (2016). Short-term Cold Acclimation Recruits Brown Adipose Tissue in Obese Humans. Diabetes.

[7] Blondin DP (2016). Four-week cold acclimation in adult humans shifts uncoupling thermogenesis from skeletal muscles to brown adipose tissue. J. Physiol..

[8] Osuna-Prieto FJ (2019). Activation of Human Brown Adipose Tissue by Capsinoids, Catechins, Ephedrine, and Other Dietary Components: A Systematic Review. Adv. Nutr..

[9] Ruiz JR (2018). Role of Human Brown Fat in Obesity, Metabolism and Cardiovascular Disease: Strategies to Turn Up the Heat. Prog. Cardiovasc. Dis..

[10] Elrick H (1996). Exercise is medicine. Phys. Sportsmed..

[11] Portugal EMM (2013). Neuroscience of exercise: From neurobiology mechanisms to mental health. Neuropsychobiology.

[12] Pedersen BK, Saltin B (2015). Exercise as medicine - Evidence for prescribing exercise as therapy in 26 different chronic diseases. Scand. J. Med. Sci. Sport..

[13] Ruiz JR, Martinez-Tellez B, Sanchez-Delgado G, Aguilera CM, Gil A (2015). Regulation of energy balance by brown adipose tissue: at least three potential roles for physical activity. Br. J. Sports Med..

[14] Sanchez-Delgado, G. *et al*. Role of exercise in the activation of brown adipose tissue. *Ann*. *Nutr*. *Metab*. **67** (2015). 10.1159/000437173 26227180

[15] Carobbio S, Guénantin AC, Vidal-Puig A (2018). ‘Basic and Applied Thermogenesis Research’ Bridging the Gap. Trends Endocrinol. Metab..

[16] Vosselman, M. J. *et al*. Low brown adipose tissue activity in endurance trained compared to lean sedentary men. *Int*. *J*. *Obes*. *(Lond)*. 1–7, 10.1038/ijo.2015.130 (2015). 10.1038/ijo.2015.130 26189600

[17] Singhal V (2016). Effect of Chronic Athletic Activity on Brown Fat in Young Women. PLoS One.

[18] Norheim F (2014). The effects of acute and chronic exercise on PGC-1α, irisin and browning of subcutaneous adipose tissue in humans. FEBS J..

[19] Motiani P (2017). Decreased insulin-stimulated brown adipose tissue glucose uptake after short-term exercise training in healthy middle-aged men. Diabetes, Obes. Metab..

[20] Dinas, P. C. *et al*. Association between habitual physical activity and brown adipose tissue activity in individuals undergoing PET-CT scan. *Clin*. *Endocrinol*. *(Oxf)*. 1–8, 10.1111/cen.12620 (2014). 10.1111/cen.12620 25262810

[21] Ruiz, J. R., Sánchez-Delgado, G., Martínez-Téllez, B., Aguilera, C. M. & Gil, A. RE: Association between habitual physical activity and brown adipose tissue activity in individuals undergoing PET-CT scan. *Clin*. *Endocrinol*. *(Oxf)*. **83** (2015). 10.1111/cen.12703 25521222

[22] Acosta FM (2018). Association of objectively measured physical activity with brown adipose tissue volume and activity in young adults. J. Clin. Endocrinol. Metab..

[23] Fiuza-Luces C (2018). Exercise benefits in cardiovascular disease: beyond attenuation of traditional risk factors. Nat. Rev. Cardiol..

[24] Ortega FB, Silventoinen K, Tynelius P, Rasmussen F (2012). Muscular strength in male adolescents and premature death: Cohort study of one million participants. BMJ.

[25] Artero LD, Xuemei Sui EG, Blair SN (2010). Review: Mortality trends in the general population: the importance of cardiorespiratory fitness. J. Psychopharmacol..

[26] Kodama S (2009). Cardiorespiratory fitness as a quantitative predictor of all-cause mortality and cardiovascular events in healthy men and women: a meta-analysis. JAMA.

[27] Henriksson, H., Henriksson, P., Tynelius, P. & Ortega, F. B. Muscular weakness in adolescence is associated with disability 30 years later: a population-based cohort study of 1.2 million men. *Br*. *J*. *Sports Med*. bjsports-2017-098723, 10.1136/bjsports-2017-098723 (2018). 10.1136/bjsports-2017-098723 29921654

[28] Sanchez-Delgado, G. *et al*. Activating brown adipose tissue through exercise (ACTIBATE) in young adults: Rationale, design and methodology. *Contemp*. *Clin*. *Trials* **45** (2015). 10.1016/j.cct.2015.11.004 26546068

[29] Sanchez-Delgado G (2017). Reliability of resting metabolic rate measurements in young adults: Impact of methods for data analysis. Clin. Nutr..

[30] Midgley AW, McNaughton LR, Polman R, Marchant D (2007). Criteria for determination of maximal oxygen uptake: A brief critique and recommendations for future research. Sport. Med..

[31] Ruiz-Ruiz J, Mesa JLM, Gutiérrez A, Castillo MJ (2002). Hand size influences optimal grip span in women but not in men. J. Hand Surg. Am..

[32] Wood TM, Maddalozzo GF, Harter RA (2002). Accuracy of seven equations for predicting 1-RM performance of apparently healthy, sedentary older adults. Meas. Phys. Educ. Exerc. Sci..

[33] Martinez-Tellez B (2018). The impact of using BARCIST 1.0 criteria on quantification of BAT volume and activity in three independent cohorts of adults. Sci. Rep..

[34] Martinez-Tellez B (2017). A New Personalized Cooling Protocol to Activate Brown Adipose Tissue in Young Adults. Front. Physiol..

[35] Chen KY (2016). Brown Adipose Reporting Criteria in Imaging STudies (BARCIST 1.0): Recommendations for Standardized FDG-PET/CT Experiments in Humans. Cell Metab..

[36] Blondin DP (2015). Contributions of white and brown adipose tissues and skeletal muscles to acute cold-induced metabolic responses in healthy men. J. Physiol..

[37] Martinez-Tellez, B. *et al*. Evidence of high 18F-Fluorodeoxyglucose uptake in the subcutaneous adipose tissue of the dorsocervical area in young adults. *Exp*. *Physiol*. 1–6, 10.1113/EP087428 (2018). 10.1113/EP087428 30468689

[38] Lehnig AC, Stanford KI (2018). Exercise-induced adaptations to white and brown adipose tissue. J. Exp. Biol..

[39] Kraemer WJ, Deschenes MR, Fleck SJ (1988). Physiological Adaptations to Resistance Exercise: Implications for Athletic Conditioning. Sport. Med. An Int. J. Appl. Med. Sci. Sport Exerc..

[40] Welinder C (2017). Physiological adaptations to resistance exercise as a function of age. J Proteome Res.

[41] Rustaden AM, Gjestvang C, Bø K, Hagen Haakstad LA, Paulsen G (2018). BodyPump versus traditional heavy load resistance training on changes in resting metabolic rate in overweight untrained women. J. Sports Med. Phys. Fitness.

[42] Thomas EM, Sahlberg M, Svantesson U (2008). The effect of resistance training on handgrip strength in young adults. Isokinet. Exerc. Sci..

[43] Moliner-Urdiales D (2011). Associations of muscular and cardiorespiratory fitness with total and central body fat in adolescents: the HELENA study. Br. J. Sports Med..

[44] Ortega, F. B. *et al*. Systematic Review and Proposal of a Field-Based Physical Fitness-Test Battery in Preschool Children: The PREFIT Battery. *Sport*. *Med*. **45** (2015). 10.1007/s40279-014-0281-8 25370201

[45] Cannon B, Nedergaard J (2004). Brown adipose tissue: function and physiological significance. Physiol. Rev..

[46] Dinas PC (2017). Browning formation markers of subcutaneous adipose tissue in relation to resting energy expenditure, physical activity and diet in humans. Horm. Mol. Biol. Clin. Investig..

[47] Hoeke G, Kooijman S, Boon MR, Rensen PCN, Berbeé JFP (2016). Role of Brown Fat in Lipoprotein Metabolism and Atherosclerosis. Circ. Res..

[48] Chondronikola M, Beeman S, Wahl RL (2017). Non-invasive methods for the assessment of brown adipose tissue in humans. J. Physiol..

