# Supplementary material for: Relationships between cardiorespiratory fitness/muscular strength and 18F-fluorodeoxyglucose uptake in brown adipose tissue after exposure to cold in young, sedentary adults
Source: Sci Rep. 2019 Aug 5;9:11314. doi: 10.1038/s41598-019-47918-7 (PMC6683147; doi:10.1038/s41598-019-47918-7)
Supplement: Supplementary file 1 — Extra analyses [file 41598_2019_47918_MOESM1_ESM.docx]

**Relationships between cardiorespiratory fitness/muscular strength and ^18^F-fluorodeoxyglucose uptake in brown adipose tissue after exposure to cold in young, sedentary adults**

**Authors´ Names:** Borja Martinez-Tellez^1,2*^, Guillermo Sanchez-Delgado^1^, Francisco J. Amaro-Gahete^1,3^, Francisco M. Acosta^1^, Jonatan R. Ruiz^1^

**Author Affiliations:** ^1^PROFITH (PROmoting FITness and Health through Physical Activity) Research Group, Sport and Health University Research Institute (iMUDS), Department of Physical Education and Sport, Faculty of Sport Sciences, University of Granada, Granada, Spain.

^2^Department of Medicine, division of Endocrinology, and Einthoven Laboratory for Experimental Vascular Medicine, Leiden University Medical Center, Leiden, The Netherlands.

^3^Department of Medical Physiology, School of Medicine, University of Granada, Granada, Spain.

**SUPPLEMENTARY FILES**

**Table S1.** Associations between muscular fitness results relative to body weight and brown adipose tissue (BAT) variables.

|  | BAT volume (ml) | | | BAT SUVmean | | | BAT SUVpeak | | |
| --- | --- | --- | --- | --- | --- | --- | --- | --- | --- |
|  | β | R2 | P | β | R2 | P | β | R2 | P |
| MODEL 1 |  |  |  |  |  |  |  |  |  |
| Handgrip strength/BM (kg) | 12.497 | 0.000 | 0.836 | 0.865 | 0.002 | 0.658 | 4.485 | 0.002 | 0.599 |
| Leg press/BM (kg) | -3.311 | 0.001 | 0.695 | -0.478 | 0.026 | 0.080 | -1.201 | 0.009 | 0.314 |
| Bench press/BM (kg) | 13.655 | 0.001 | 0.706 | -1.726 | 0.019 | 0.140 | -4.333 | 0.006 | 0.397 |
| MODEL 2 |  |  |  |  |  |  |  |  |  |
| Handgrip strength/BM (kg) | 6.877 | 0.170 | 0.901 | 0.701 | 0.139 | 0.701 | 3.740 | 0.151 | 0.636 |
| Leg press/BM (kg) | -3.198 | 0.171 | 0.679 | -0.474 | 0.164 | 0.062 | -1.186 | 0.158 | 0.283 |
| Bench press/BM (kg) | 4.471 | 0.170 | 0.893 | -2.003 | 0.163 | 0.066 | -5.574 | 0.159 | 0.240 |
| MODEL 3 |  |  |  |  |  |  |  |  |  |
| Handgrip strength/BM (kg) | -29.177 | 0.207 | 0.605 | 1.534 | 0.158 | 0.416 | 5.531 | 0.155 | 0.502 |
| Leg press/BM (kg) | -20.526 | 0.238 | **0.028** | -0.399 | 0.165 | 0.205 | -1.192 | 0.158 | 0.386 |
| Bench press/BM (kg) | -101.906 | 0.237 | **0.029** | -1.830 | 0.163 | 0.245 | -7.153 | 0.160 | 0.299 |

Model 1: Unadjusted. Model 2: adjusted by date when positron emission tomography/computed tomography (PET/CT) was performed. Model 3: adjusted by date of PET/CT and sex. Model 4: adjusted by date of PET/CT. sex and body mass index. β=non-standardised coefficients; R^2^=explained variance. BM= body mass. n=119 subjects.

**Table S2.** Associations between cardiorespiratory fitness variables and skeletal muscle, dorsocervical and reference tissue (descending aorta) ^18^F-FDG uptakes.

|  | Deep skeletal muscle SUVpeak | | | Superficial skeletal muscle SUVpeak | | | Cold-sensitive skeletal muscle SUVpeak | | | Mean skeletal muscle SUVpeak | | | Dorsocervical WAT SUVpeak | | | Descending aorta SUVpeak | | |
| --- | --- | --- | --- | --- | --- | --- | --- | --- | --- | --- | --- | --- | --- | --- | --- | --- | --- | --- |
|  | β | R2 | P | β | R2 | P | β | R2 | P | β | R2 | P | β | R2 | P | β | R2 | P |
| MODEL 1 |  |  |  |  |  |  |  |  |  |  |  |  |  |  |  |  |  |  |
| Time to exhaustion (s) | -0.001 | 0.038 | 0.055 | -0.001 | 0.113 | **0.001** | -0.001 | 0.026 | 0.110 | 0.000 | 0.032 | 0.080 | 0.000 | 0.003 | 0.571 | 0.000 | 0.000 | 0.833 |
| VO_2_max (ml/kg LBM/min) | 0.002 | 0.001 | 0.694 | -0.003 | 0.008 | 0.353 | 0.004 | 0.004 | 0.477 | 0.000 | 0.001 | 0.792 | 0.000 | 0.000 | 0.815 | -0.002 | 0.015 | 0.190 |
| MODEL 2 |  |  |  |  |  |  |  |  |  |  |  |  |  |  |  |  |  |  |
| Time to exhaustion (s) | -0.001 | 0.056 | **0.041** | -0.001 | 0.113 | **0.001** | -0.001 | 0.035 | 0.093 | 0.000 | 0.036 | 0.093 | 0.000 | 0.004 | 0.566 | 0.000 | 0.007 | 0.776 |
| VO_2_max (ml/kg LBM/min) | 0.001 | 0.034 | 0.916 | -0.003 | 0.009 | 0.335 | 0.003 | 0.019 | 0.605 | 0.000 | 0.016 | 0.948 | 0.000 | 0.003 | 0.881 | -0.002 | 0.015 | 0.208 |
| MODEL 3 |  |  |  |  |  |  |  |  |  |  |  |  |  |  |  |  |  |  |
| Time to exhaustion (s) | -0.001 | 0.061 | 0.076 | -0.001 | 0.120 | **0.003** | 0.000 | 0.047 | 0.178 | 0.000 | 0.068 | 0.245 | 0.000 | 0.045 | 0.918 | 0.000 | 0.237 | 0.273 |
| VO_2_max (ml/kg LBM/min) | 0.000 | 0.055 | 0.968 | -0.003 | 0.039 | 0.253 | 0.002 | 0.044 | 0.718 | 0.000 | 0.049 | 0.805 | 0.000 | 0.029 | 0.788 | -0.002 | 0.191 | 0.355 |
| Model 1: Unadjusted. Model 2: adjusted by date when positron emission tomography/computed tomography (PET/CT) was performed. Model 3: adjusted by date of PET/CT and sex. Model 4: adjusted by date of PET/CT. sex and body mass index. β=non-standardised coefficients; R^2^=explained variance. WAT=white adipose tissue. n=98 subjects. | | | | | | | | | | | | | | | | | | |

**Table S3.** Associations of muscular strength (relative to lean body mass) with skeletal muscles, dorsocervical white adipose tissue and descending aorta glucose uptake outcomes in 119 young healthy adults.

|  | Deep skeletal muscles (SUVpeak) | | | Superficial skeletal muscles (SUVpeak) | | | Cold sensitivity skeletal muscles (SUVpeak) | | | Average skeletal muscles (SUVpeak) | | | Dorsocervical (WAT) (SUVpeak) | | | Descending aorta (SUVpeak) | | |
| --- | --- | --- | --- | --- | --- | --- | --- | --- | --- | --- | --- | --- | --- | --- | --- | --- | --- | --- |
|  | β | R2 | P | β | R2 | P | β | R2 | P | β | R2 | P | β | R2 | P | β | R2 | P |
| MODEL 1 |  |  |  |  |  |  |  |  |  |  |  |  |  |  |  |  |  |  |
| Handgrip strength/LBM (kg) | 0.199 | 0.005 | 0.459 | -0.119 | 0.007 | 0.350 | 0.293 | 0.009 | 0.293 | 0.028 | 0.000 | 0.871 | 0.008 | 0.000 | 0.972 | -0.254 | 0.006 | 0.386 |
| Leg press/LBM (kg) | 0.008 | 0.000 | 0.821 | 0.006 | 0.001 | 0.706 | -0.011 | 0.001 | 0.758 | 0.000 | 0.000 | 0.989 | 0.003 | 0.000 | 0.901 | 0.036 | 0.008 | 0.331 |
| Bench press/LBM (kg) | -0.020 | 0.000 | 0.884 | -0.100 | 0.020 | 0.122 | -0.022 | 0.000 | 0.877 | -0.083 | 0.007 | 0.353 | -0.093 | 0.006 | 0.395 | 0.075 | 0.002 | 0.614 |
| MODEL 2 |  |  |  |  |  |  |  |  |  |  |  |  |  |  |  |  |  |  |
| Handgrip strength/LBM (kg) | 0.170 | 0.035 | 0.525 | -0.118 | 0.008 | 0.356 | 0.273 | 0.022 | 0.327 | 0.013 | 0.019 | 0.940 | 0.003 | 0.003 | 0.990 | -0.252 | 0.007 | 0.393 |
| Leg press/LBM (kg) | 0.007 | 0.032 | 0.842 | 0.006 | 0.001 | 0.706 | -0.012 | 0.015 | 0.743 | 0.000 | 0.019 | 0.993 | 0.004 | 0.003 | 0.898 | 0.036 | 0.008 | 0.331 |
| Bench press/LBM (kg) | -0.041 | 0.033 | 0.762 | -0.100 | 0.020 | 0.126 | -0.037 | 0.015 | 0.796 | -0.094 | 0.028 | 0.291 | -0.098 | 0.010 | 0.371 | 0.078 | 0.003 | 0.605 |
| MODEL 3 |  |  |  |  |  |  |  |  |  |  |  |  |  |  |  |  |  |  |
| Handgrip strength/LBM (kg) | 0.169 | 0.035 | 0.527 | -0.117 | 0.008 | 0.361 | 0.277 | 0.025 | 0.321 | 0.015 | 0.020 | 0.933 | -0.001 | 0.008 | 0.995 | -0.269 | 0.044 | 0.354 |
| Leg press/LBM (kg) | 0.008 | 0.032 | 0.841 | 0.010 | 0.003 | 0.594 | -0.004 | 0.016 | 0.920 | 0.004 | 0.020 | 0.884 | -0.008 | 0.009 | 0.797 | 0.000 | 0.037 | 0.995 |
| Bench press/LBM (kg) | -0.085 | 0.034 | 0.649 | -0.169 | 0.032 | 0.058 | 0.024 | 0.017 | 0.902 | -0.142 | 0.031 | 0.244 | -0.299 | 0.043 | 0.047 | -0.251 | 0.050 | 0.215 |
| Model 1: Unadjusted. Model 2: adjusted by date when positron emission tomography/computed tomography (PET/CT) was performed. Model 3: adjusted by date of PET/CT and sex. β=non-standardised coefficients; BM=body mass; R^2^=explained variance; SUV=standardised uptake value; LBM: lean body mass; VO_2_max=maximum volume of oxygen consumed. WAT=white adipose tissue. | | | | | | | | | | | | | | | | | | |
